# Novel Role of Endogenous Catalase in Macrophage Polarization in Adipose Tissue

**DOI:** 10.1155/2016/8675905

**Published:** 2016-08-15

**Authors:** Ye Seul Park, Md Jamal Uddin, Lingjuan Piao, Inah Hwang, Jung Hwa Lee, Hunjoo Ha

**Affiliations:** Graduate School of Pharmaceutical Sciences, College of Pharmacy, Ewha Womans University, 52 Ewhayeodae-gil, Seodaemun-gu, Seoul 120-750, Republic of Korea

## Abstract

Macrophages are important components of adipose tissue inflammation, which results in metabolic diseases such as insulin resistance. Notably, obesity induces a proinflammatory phenotypic switch in adipose tissue macrophages, and oxidative stress facilitates this switch. Thus, we examined the role of endogenous catalase, a key regulator of oxidative stress, in the activity of adipose tissue macrophages in obese mice. Catalase knockout (CKO) exacerbated insulin resistance, amplified oxidative stress, and accelerated macrophage infiltration into epididymal white adipose tissue in mice on normal or high-fat diet. Interestingly, catalase deficiency also enhanced classical macrophage activation (M1) and inflammation but suppressed alternative activation (M2) regardless of diet. Similarly, pharmacological inhibition of catalase activity using 3-aminotriazole induced the same phenotypic switch and inflammatory response in RAW264.7 macrophages. Finally, the same phenotypic switch and inflammatory responses were observed in primary bone marrow-derived macrophages from CKO mice. Taken together, the data indicate that endogenous catalase regulates the polarization of adipose tissue macrophages and thereby inhibits inflammation and insulin resistance.

## 1. Introduction

Excess body weight and obesity have become worldwide epidemics, resulting in detrimental health problems including hypertension, atherosclerosis, cancer, type 2 diabetes mellitus, and insulin resistance [1]. The development and progression of these diseases are linked to chronic and low-grade inflammation in adipose tissue [2–5]. In turn, inflammation is promoted or resolved by macrophages, which polarize into classically (M1) or alternatively (M2) activated macrophages depending on environmental stimuli [5–7]. Accordingly, macrophages have been proposed as components of adipose tissue inflammation [8, 9].

Obesity not only increases the number of adipose tissue macrophages but also induces a phenotypic switch [10]. In lean adipose tissue, resident macrophages are polarized toward M2 and thus express F4/80, CD301, CD206, interleukin-10 (IL-10), and arginase-1 [11, 12]. M2 macrophages help maintain adipose tissue function by preventing inflammation and promoting insulin sensitivity [13]. In contrast, macrophages in obese adipose tissue are predominantly M1 and express F4/80, CD11c, tumor necrosis factor-*α* (TNF-*α*), IL-6, and inducible nitric oxide synthase (iNOS) [11]. These M1 macrophages also secrete proinflammatory cytokines that interfere with insulin signaling and form crown-like structures to phagocytize dead adipocytes [14–19]. In addition, excess glucose and fatty acids in obese adipose tissue may increase the mitochondrial electron flux and impair mitochondrial respiratory capacity, resulting in an overload of reactive oxygen species (ROS) [20]. Oxidative stress due to these species then accelerates insulin resistance, promotes the development metabolic disorders [21], and induces the phenotypic switch in adipose tissue macrophages [22].

Catalase is a well-recognized antioxidant enzyme, and overexpression protects transgenic mice against inflammation-associated injury, including atherosclerosis [23] and diabetic kidney disease [24, 25]. In addition, forced overexpression of catalase in mitochondria protects insulin-producing *β*-cells against injury from ROS and against toxicity from proinflammatory cytokines [26]. On the other hand, catalase deficiency induces oxidant-mediated tissue injury [27], increases mitochondrial ROS in mice with excess body weight, and accelerates diabetic kidney injury [28].

However, the relationship between catalase and macrophage activity is not well characterized. In J774.16 macrophages, exogenous catalase reduces nitric oxide production in response to lipopolysaccharides (LPS) and interferon-*γ* [29]. In human alveolar macrophages, exposure to 3-aminotriazole, a catalase inhibitor, increases basal H_2_O_2_, activates p38 MAP kinase, and elicits inflammatory responses [30]. However, the role of macrophage catalase in obesity-induced inflammation of the adipose tissue remains to be studied. Thus, we used macrophage cultures and a mouse model to examine the effects of catalase deficiency on the polarization of adipose tissue macrophages. We found that catalase deficiency increases the M1/M2 ratio in mice on both normal diet (ND) and high-fat diet (HFD) and thereby enhances inflammation and insulin resistance in adipose tissue.

## 2. Materials and Methods

### 2.1. Reagents and Chemicals

Dulbecco's Modified Eagle Medium (DMEM) and penicillin-streptomycin were purchased from Life Technologies (Carlsbad, CA, USA). Fetal bovine serum (FBS) was obtained from Thermo Fisher Scientific (Waltham, MA, USA). All other chemicals were obtained from Sigma-Aldrich unless otherwise noted.

### 2.2. Animals

Animals were housed at 22 ± 2°C on a 12-hour dark/12-hour light cycle and were provided with tap water* ad libitum* unless indicated otherwise. Male catalase wild type (WT) and catalase knockout (CKO) C57BL/6 J mice [27] were provided by Professors Rhee and Woo at Ewha Womans University. Mice were fed for 21 weeks with either ND or HFD. In ND, 28%, 54%, and 18% of calories are derived from protein, carbohydrate, and fat, respectively (Harlan Teklad 2018S, Indianapolis, IN, USA), while 18.4%, 21.3%, and 60.3% of calories in HFD are derived from protein, carbohydrate, and fat, respectively (Harlan TD06414). Plasma insulin was measured in blood collected from the orbital sinus a day before sacrifice at 21 weeks. For* in vivo* insulin stimulation and analysis of insulin signaling in adipose tissue, mice were fasted overnight and anesthetized with 16.5% urethane (10 mL/kg). Abdominal cavities were opened, and the right section of epididymal white adipose tissue (WAT) was rapidly excised for analysis of insulin signaling. Mice were then injected via the inferior vena cava with 10 U/kg Humulin® R (Eli Lilly, Indianapolis, IN, USA). All animal experiments were approved by the Institutional Animal Care and Use Committee at Ewha Womans University (number 2013-01-011).

### 2.3. Blood Parameters

Blood samples were centrifuged at 3,000 rpm for 15 min at 4°C, and plasma in the supernatant was collected. Plasma triglycerides, low-density lipoprotein (LDL)/very low-density lipoprotein (VLDL), high-density lipoprotein (HDL), and free fatty acids were measured using EnzyChrom*™* colorimetric assay kits (BioAssay Systems, Hayward, CA, USA) according to the manufacturer's instructions. Commercial ELISA kits (R&D Systems) were used to measure plasma insulin following the manufacturer's protocol.

### 2.4. Isolation of Stromal Vascular Fraction

Epididymal fat pads were collected and minced in phosphate-buffered saline (PBS) supplemented with 1% bovine serum albumin and 0.2 mg/mL DNase I. Tissues were then digested with 2 mg/mL collagenase for 20 min at 37°C and 400 rpm shaking, filtered through 100 *μ*m, and centrifuged at 300 ×g for 5 min to separate floating adipocytes from the pellet [11].

### 2.5. Preparation of PA

PA was dissolved in 50% ethanol, clarified by heating to 60°C, and added dropwise to warmed 10% fatty acid-free bovine serum albumin (45~52°C) dissolved in PBS. The pH of the mixture was adjusted to 7.0~7.4 with NaOH, and aliquots were frozen and stored at −20°C.

### 2.6. Bone Marrow-Derived Macrophages

After euthanasia, 8-week-old C57BL/6 mice were sprayed with 70% ethanol, and femurs were dissected using scissors, cutting through the tibia below the knee joints, as well as through the pelvic bone close to the hip joint. Muscles connected to the bone were removed using clean gauze, and femurs were placed on ice in polypropylene tubes containing sterile PBS. Fresh bone marrow cells [31] were obtained by aseptically flushing bones with a syringe filled with RPMI1640 medium. Cells were resuspended in 10 mL RPMI1640 medium supplemented with 10% fetal bovine serum, 20 ng/mL macrophage colony stimulating factor, 100 U/mL penicillin, and 100 *μ*g/mL streptomycin. Cells were then seeded in non-tissue culture treated Petri dishes and incubated at 37°C in 5% CO_2_. Media were refreshed four days later, and cultures were incubated for an additional three days. Bone marrow-derived macrophages attached to plates were harvested with trypsin (Gibco) and treated with or without PA or IL-4 for 6 h.

### 2.7. Culture of RAW264.7 Macrophages

RAW264.7 cells were procured from American Type Culture Collection (Manassas, VA, USA) and maintained at 37°C in humidified air with 5% CO_2_ and in DMEM supplemented with 10% fetal bovine serum, 100 U/mL penicillin, 100 *μ*g/mL streptomycin, and 44 mM NaHCO_3_. Cells were then grown to 60–70% confluence in 6-well plates, incubated with low-serum media for 24 h, and treated with 3-aminotriazole (3-AT) for 6 h or 24 h to inactivate catalase. The inhibitor was preincubated for 1 h in LPS or for 6 h or 24 h in PA and bovine serum albumin (BSA). Pure BSA at 0.3% was used to control for the effects of PA.

### 2.8. Immunohistochemistry

A commercially available kit based on immunoperoxidase was used for immunohistochemistry (Dako, Glostrup, Denmark). Briefly, epididymal adipose tissues from WT and CKO mice on ND or HFD were fixed in 10% formalin, dehydrated, embedded in paraffin wax, and subsequently deparaffinized. Endogenous peroxidase was quenched for 30 min using Dako peroxidase solution, and tissue sections were then washed and blocked with Dako serum-free blocking solution. Sections were subsequently probed overnight at 4°C with antibodies against F4/80 (1 : 100, Santa Cruz Biotechnology Inc., Santa Cruz, CA, USA), CD11c (1 : 200, Santa Cruz Biotechnology Inc.), CD206 (1 : 50, Santa Cruz Biotechnology Inc.), and nitrotyrosine (1 : 200, Santa Cruz Biotechnology Inc.). After washing in phosphate-buffered saline, sections were processed with a LSAB2 Kit (Dako) and labeled with 3,3′-diaminobenzidine for 4 min. Digital images were captured on a Zeiss microscope equipped with an Axio Cam HRC digital camera and software (Carl Zeiss, Thornwood, NY, USA) and analyzed with the open-source program ImageJ v1.34s (Rasband, WS, ImageJ, US National Institutes of Health, Bethesda, MD, USA).

### 2.9. Western Blot

Adipose tissue homogenates or harvested cells were analyzed by western blot to assess phosphorylation of c-Jun N-terminal kinase (JNK) at Thr183 and Tyr185, as well as expression of t-JNK and iNOS [28]. Briefly, adipose tissues were lysed in TGN buffer and centrifuged at 13,000 rpm and 4°C for 15 min. The protein concentration in lysates was determined by Bradford method (Bio-Rad Laboratories, Hercules, CA, USA). Samples of tissues and cell extracts were mixed with buffer containing SDS and mercaptoethanol, heated at 95°C for 5 min, resolved by SDS-PAGE, and transferred onto a polyvinylidene fluoride membrane (GE Healthcare BioSciences Co., Piscataway, NJ, USA) in a transblot chamber with Tris buffer. The membrane was blocked for 1 h at room temperature with 5% skim milk in TBS buffer supplemented with Tween 20 and probed overnight at 4°C with polyclonal antibodies against phosphorylated JNK (1 : 1000, Cell Signaling Technology), t-JNK (1 : 2000, Cell Signaling Technology), iNOS (1 : 2000, Santa Cruz), and *β*-actin (1 : 2000, Santa Cruz). Membranes were then washed, labeled for 1 h at room temperature with peroxidase-conjugated secondary antibody, washed another few times, and visualized with enhanced chemiluminescence detection reagent (GE Healthcare BioSciences Co.) according to the manufacturer's instructions. Immunoreactive bands were quantified by densitometry using ImageJ software and normalized to t-JNK and *β*-actin.

### 2.10. Real-Time RT-PCR

Real-time RT-PCR was used to measure expression of arginase-1, CD11c, CD206, F4/80, IL-1*β*, IL-6, IL-10, plasminogen activator inhibitor (PAI-1), and TNF-*α*. Total RNA was extracted from tissues and cells using TRIzol (Life Technologies), and cDNA was synthesized as previously described [28]. mRNA expression was assessed in duplicate on an ABI 7300 real-time PCR thermal cycler (Applied Biosystems, Foster City, CA, USA) using SYBR Green PCR Master Mix Kit (Applied Biosystems) in a final volume of 20 *μ*L. Amplified products were then quantified from standard curves using Applied Biosystems software, and expression was normalized to 18S mRNA. Primer sequences are listed in Table 1.

### 2.11. Statistical Analysis

Data are reported as mean ± standard error. Analysis of variance was used to compare multiple groups. If the *F* statistic was significant, means were compared by Fisher's least significant difference method. A *p* value < 0.05 was considered to indicate significance.

## 3. Results

### 3.1. Metabolic Characteristics Are Altered in CKO Mice

As expected, HFD increased most metabolic parameters in both WT and CKO mice, including body weight, epididymal fat mass, plasma triglycerides, LDL/VLDL, fed and fasting insulin, and homeostatic model assessment-insulin resistance (HOMA-IR) (Table 2). Surprisingly, plasma free fatty acids and triglycerides were significantly higher in CKO mice than in WT mice, even on ND (Table 2). Further, catalase deficiency accelerated insulin resistance due to HFD (Table 2). These results demonstrate that catalase deficiency causes systemic dyslipidemia in mice regardless of diet.

Macrophages are not the only cells regulated by catalase, but other types of cell are also regulated by catalase. In fact, mammalian catalase is the peroxisomal protein and abundantly expressed in liver and kidney [27]. In addition, we observed catalase expression in WAT (see Supplementary Figure 1 in Supplementary Material available online at http://dx.doi.org/10.1155/2016/8675905). Thus, these organs which express catalase will be affected by catalase knockdown. Since liver and WAT play key roles in lipid metabolism and insulin resistance [32–34], the functions of these organs in CKO mice must be affected as reflected by systemic dyslipidemia and insulin resistance (Table 2).

### 3.2. Catalase Deficiency Accelerates Oxidative Stress, Macrophage Infiltration, and Formation of Crown-Like Structures in Epididymal WAT

**Figure 1:**
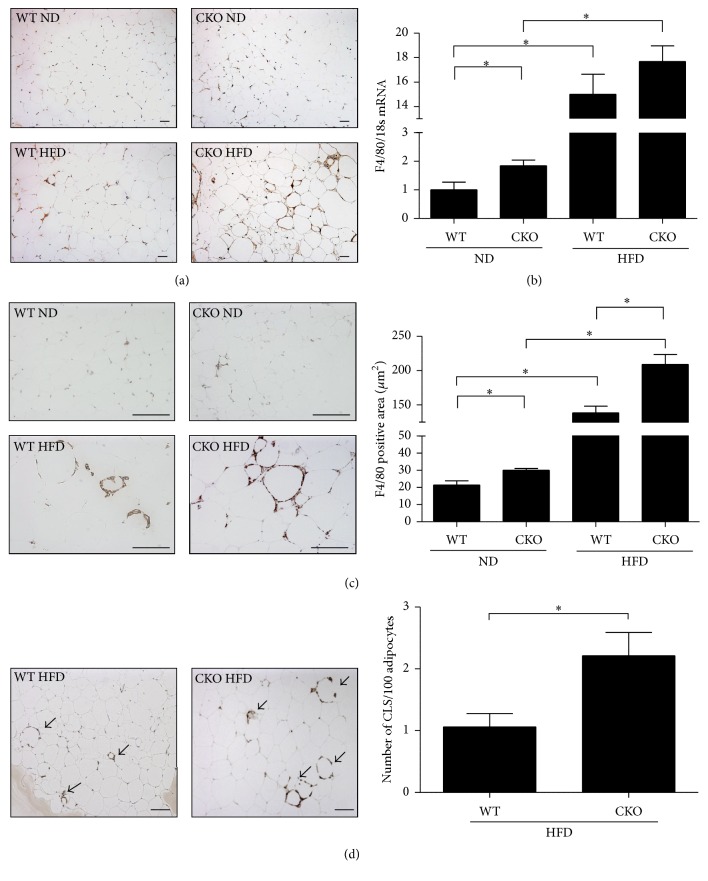
Catalase deficiency accelerates oxidative stress, macrophage infiltration, and crown-like structures in epididymal WAT. (a) Immunohistochemistry for nitrotyrosine (1 : 200), a marker of oxidative stress. Scale bar, 50 *μ*m; magnification, 100x. (b) F4/80 was assessed by real-time PCR and (c) immunohistochemistry (1 : 100) to measure macrophage infiltration. Immunostaining was quantified in Image Pro. Scale bar, 100 *μ*m; magnification, 200x. (d) Crown-like structures in M1 macrophages were also quantified by immunohistochemistry for F4/80 (1 : 100, brown) and image analysis in Image Pro. Scale bar, 100 *μ*m; magnification, 100x. Tissues were costained with hematoxylin (blue). Data are mean ± SE of 6–8 mice, and representative immunohistochemistry images are shown. ^*∗*^ *p* < 0.05.

Oxidative stress in epididymal WAT dramatically increased in CKO mice on ND (Figure 1(a)), as measured by nitrotyrosine staining [35]. HFD further amplified this effect (Figure 1(a)). Similar trends were observed in the abundance of F4/80 mRNA, a measure of macrophage infiltration into epididymal WAT (Figure 1(b)). Accordingly, F4/80 immunostaining was higher in CKO mice than in WT mice regardless of diet. In addition, F4/80 immunostaining was stronger in mice on HFD than those on ND (Figure 1(c)). Notably, few crown-like structures, which are formed by M1 macrophages around dead adipocytes, were observed on ND. However, the number of crown-like structures per 100 adipocytes increased over 2-fold in CKO mice on HFD compared to that in WT mice on the same diet (Figure 1(d)). These findings suggest that catalase inhibits macrophage infiltration and formation of crown-like structures in epididymal WAT.

### 3.3. Catalase Deficiency Alters Inflammation and Macrophage Activation in Epididymal WAT

**Figure 2:**
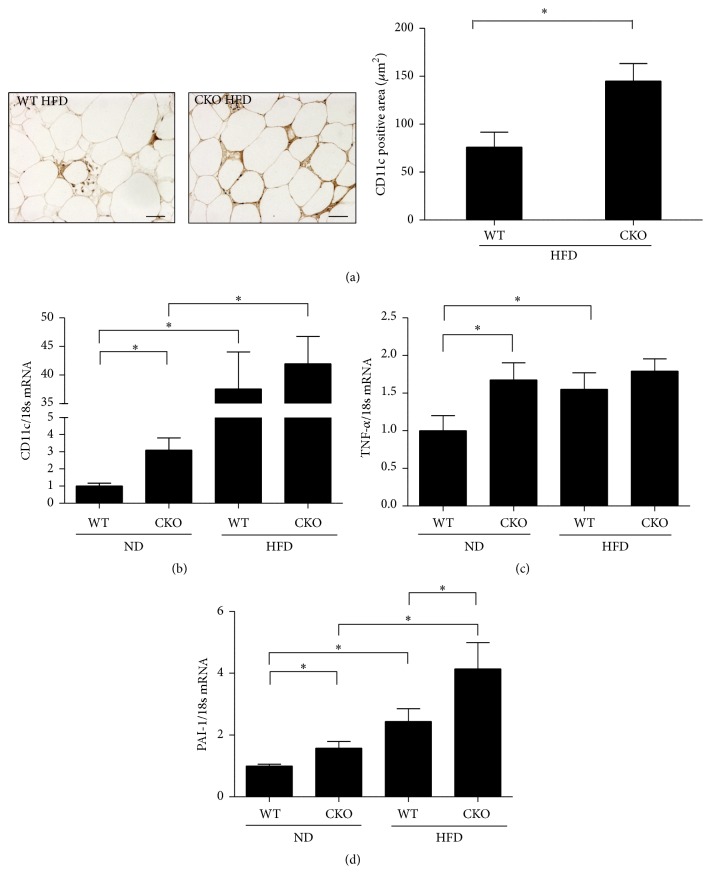
Catalase deficiency favors M1 activation and inflammation in the stromal vascular fraction of epididymal WAT. (a) To measure M1 activation, CD11c (1 : 200) was assessed by immunohistochemistry (brown) and quantified in Image Pro. Scale bar, 50 *μ*m; magnification, 200x. Samples were costained with hematoxylin (blue). (b) CD11c, (c) TNF-*α*, and (d) PAI-1 mRNA levels were determined by real-time PCR. Data are mean ± SE of 6–8 mice. ^*∗*^ *p* < 0.05.

In epididymal WAT, immunostaining for CD11c, a marker of classically activated M1 macrophages, was stronger in CKO mice on HFD than in WT mice (Figure 2(a)). To characterize macrophages more specifically, the stromal vascular fraction was obtained. This fraction contains macrophages, preadipocytes, and other immune cells, but not adipocytes. Expression of CD11c, TNF-*α*, and PAI-1 mRNA in this fraction was significantly higher in WT mice on HFD than in mice on ND (Figures 2(b)–2(d)). These markers were also more abundantly expressed in CKO mice on ND than in WT mice on the same diet (Figures 2(b)–2(d)). Further, HFD amplified expression of CD11c and PAI-1 mRNA in CKO mice (Figures 2(b) and 2(d)).

**Figure 3:**
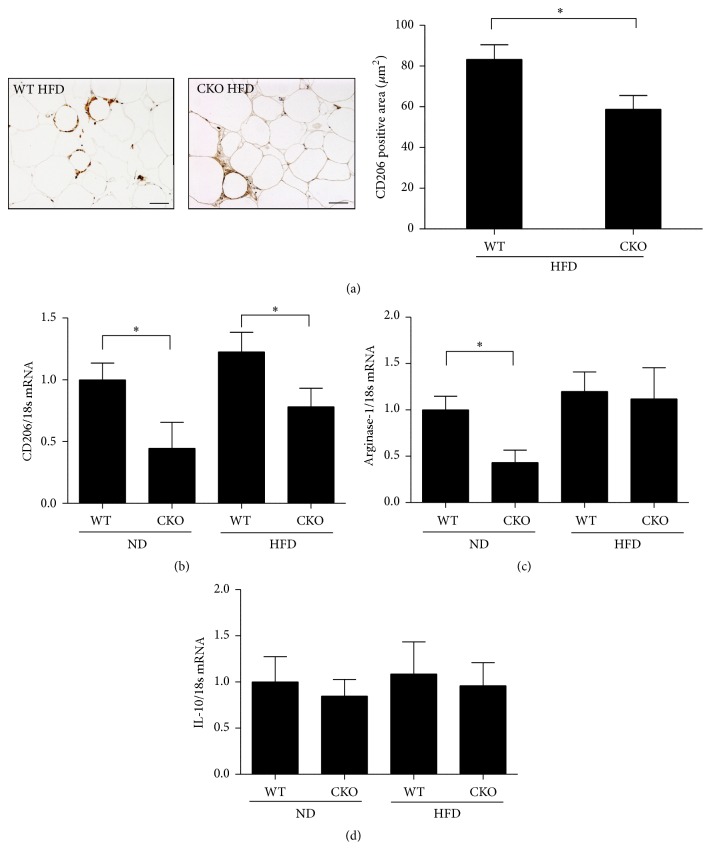
Catalase deficiency suppresses M2 macrophages in the stromal vascular fraction of epididymal WAT. (a) CD206 (1 : 20, brown) was assessed by immunohistochemistry to measure M2 activation, using hematoxylin as counterstain (blue). Images were analyzed in Image Pro. Scale bar, 50 *μ*m; magnification, 200x. (b) CD206, (c) arginase-1, and (d) IL-10 mRNA levels were determined by real-time PCR. Data are mean ± SE of 6–8 mice. ^*∗*^ *p* < 0.05.

On the other hand, catalase deficiency decreased the abundance of alternatively activated M2 macrophages, as assessed by CD206 staining of epididymal WAT from mice on HFD (Figure 3(a)). Accordingly, CKO significantly reduced CD206 mRNA by 0.45- and 0.58-fold in mice on ND and HFD, respectively, in comparison to WT (Figure 3(b)). Additionally, mRNA expression of arginase-1, an enzyme released from M2 macrophages, was decreased in CKO mice on ND compared to that in WT mice on the same diet (Figure 3(c)). However, expression of IL-10 mRNA was comparable among strains regardless of diet (Figure 3(d)). Collectively, the data indicate that catalase is a key regulator of inflammation and macrophage phenotype.

### 3.4. Genetic Knockdown of Catalase Exacerbates Palmitate-Induced Inflammation

**Figure 4:**
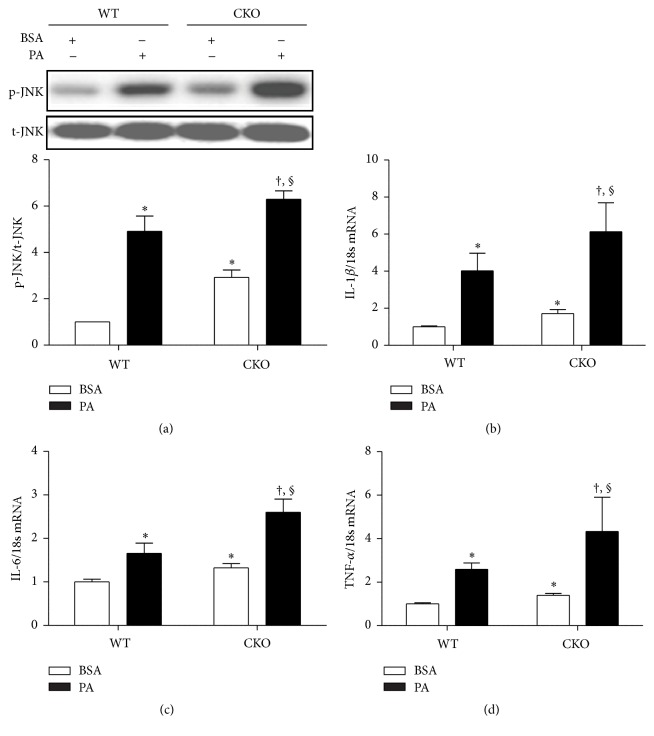
Catalase knockdown increases palmitate-induced inflammation in bone marrow-derived macrophages. Bone marrow-derived macrophages from WT and CKO mice were treated with 500 *μ*M PA for 6 h, using BSA as control. (a) JNK phosphorylation was measured by western blot (upper panel) and densitometry (lower panel). (b) IL-1*β*, (c) IL-6, and (d) TNF-*α* mRNA expression was measured by real-time PCR. Data are mean ± SE of four experiments. ^*∗*^ *p* < 0.05 versus WT macrophages exposed to BSA; ^†^ *p* < 0.05 versus CKO macrophages exposed to BSA; ^§^ *p* < 0.05 versus WT macrophages exposed to PA.

Bone marrow-derived macrophages were isolated from WT and CKO mice and exposed to 500 *μ*M PA. In comparison to WT, CKO upregulated JNK phosphorylation with or without exposure to PA (Figure 4(a)). Accordingly, mRNA levels of IL-1*β*, IL-6, and TNF-*α* also increased in CKO cells regardless of exposure to PA (Figures 4(b)–4(d)). These data demonstrate that catalase knockdown induces basal inflammation, an effect enhanced by exposure to PA.

### 3.5. Pharmacological Inhibition of Catalase Induces Inflammation in RAW264.7 Macrophages

**Figure 5:**
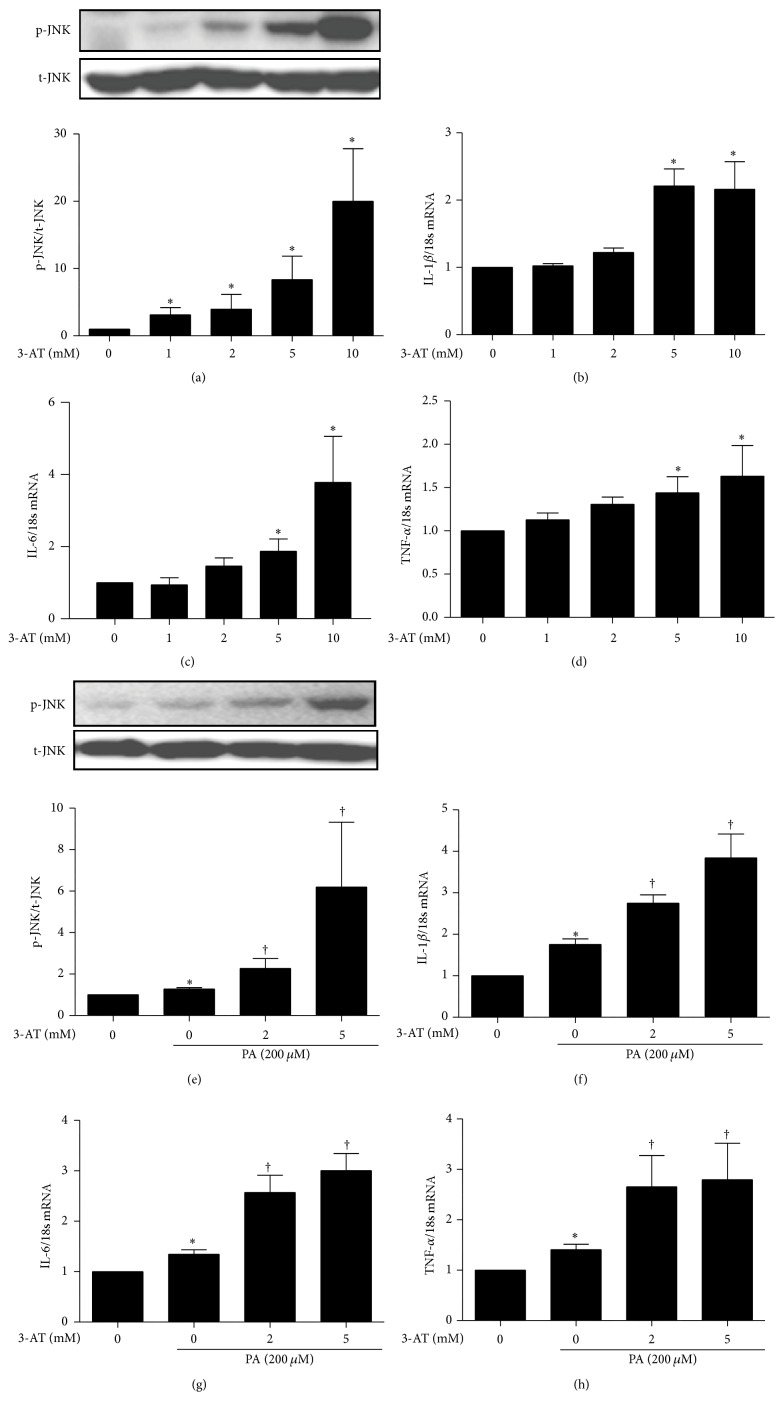
Catalase inhibition accelerates inflammation in RAW264.7 macrophages. RAW264.7 cells were treated for 6 h with 0, 1, 2, 5, and 10 mM 3-AT. (a) JNK phosphorylation was quantified by western blot (upper panel) and densitometry (lower panel). (b) IL-1*β*, (c) IL-6, and (d) TNF-*α* mRNA levels were measured by real-time PCR. (e) JNK phosphorylation was also determined by western blot (upper panel) and densitometry (lower panel) in cells treated with 0, 1, 2, 5, and 10 mM 3-AT 1 h prior to stimulation with 200 *μ*M PA for 6 h. (f) IL-1*β*, (g) IL-6, and (h) TNF-*α* mRNA levels were also measured by real-time PCR in these cells. Data are mean ± SE of four experiments. BSA was used as control for the effects of PA. ^*∗*^ *p* < 0.05 versus control; ^†^ *p* < 0.05 versus PA.

To support the genetic intervention employed* in vivo*, RAW264.7 macrophages were treated with 3-AT, a known pharmacological inhibitor of catalase. The inhibitor boosted the abundance of phosphorylated JNK in dose-dependent manner (Figure 5(a)). Notably, we found that 5 and 10 mM 3-AT upregulated the basal mRNA expression of the proinflammatory cytokines IL-1*β*, IL-6, and TNF-*α* (Figures 5(b)–5(d)). These results suggest that inhibition of catalase induces basal inflammation. Further, western blotting showed that exposure to 200 *μ*M PA induced phosphorylation (activation) of JNK. This effect was further enhanced by pretreatment with 2 or 5 mM 3-AT for 1 h (Figure 5(e)). Accordingly, pretreatment with 3-AT significantly accelerated PA-induced production of proinflammatory cytokines (Figures 5(f)–5(h)). These data indicate that catalase suppresses basal and PA-induced inflammation in murine macrophages.

### 3.6. Pharmacological or Genetic Inhibition of Catalase Alters Activation of Macrophages and Inflammation

**Figure 6:**
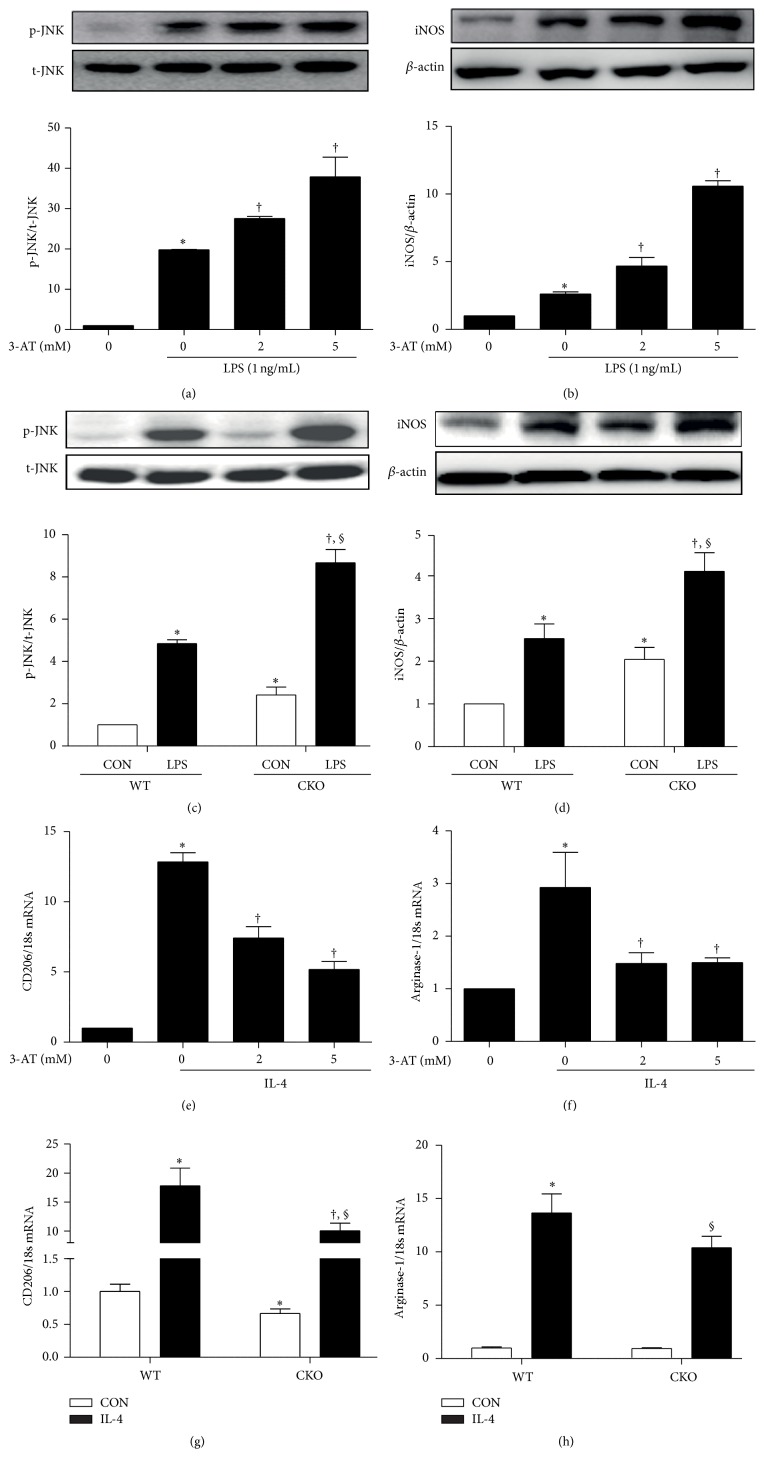
Pharmacological or genetic inhibition of catalase alters macrophage activation in response to LPS or IL-4. RAW264.7 cells were treated with 0, 1, 2, 5, and 10 mM 3-AT and stimulated with 1 ng/mL LPS for 6 h or 24 h. (a) JNK phosphorylation at 6 h and (b) iNOS levels at 24 h were quantified by western blot (upper panel) and densitometry (lower panel). ^*∗*^ *p* < 0.05 versus control; ^†^ *p* < 0.05 versus LPS. (c) JNK phosphorylation at 6 h and (d) iNOS abundance at 24 h were also measured by western blot (upper panel) and densitometry (lower panel) in WT and CKO bone marrow-derived macrophages treated with 1 ng/mL LPS for 6 h or 24 h. ^*∗*^ *p* < 0.05 versus unstimulated WT macrophages; ^†^ *p* < 0.05 versus unstimulated CKO macrophages; ^§^ *p* < 0.05 versus WT macrophages exposed to LPS. All data are mean ± SE of four experiments. (e) CD206 and (f) arginase-1 mRNA levels were measured in RAW264.7 cells treated with 0, 2, and 5 mM 3-AT 1 h prior to stimulation with 10 ng/mL IL-4 for 6 h. ^*∗*^ *p* < 0.05 versus control; ^†^ *p* < 0.05 versus IL-4. (g) CD206 and (h) arginase-1 mRNA levels were also measured in WT and CKO bone marrow-derived macrophages treated with 10 ng/mL IL-4 for 6 h. ^*∗*^ *p* < 0.05 versus unstimulated WT macrophages; ^†^ *p* < 0.05 versus unstimulated CKO macrophages; ^§^ *p* < 0.05 versus WT macrophages exposed to IL-4. Data are from real-time PCR and are reported as mean ± SE of four experiments.

In RAW264.7 macrophages exposed to 3-AT and in primary bone marrow-derived macrophages from CKO mice, classical macrophage activation was enhanced under basal (Figures 5(a)–5(d) and 6(c)) and LPS-induced conditions, as measured by JNK phosphorylation (Figure 6(a)) and iNOS expression (Figures 6(b)–6(d)). On the other hand, 2 and 5 mM 3-AT reduced alternative activation of RAW264.7 macrophages in response to IL-4, as measured by CD206 and arginase-1 mRNA (Figures 6(e) and 6(f)). Similarly, IL-4-induced alternative activation was repressed in bone marrow-derived macrophages from CKO mice compared to that in WT macrophages (Figures 6(g) and 6(h)). Notably, basal levels of CD206 mRNA were significantly reduced in CKO macrophages compared to those in WT (Figure 3(b)), in agreement with* in vivo* data. Taken together, the data imply that pharmacological or genetic inhibition of catalase activity favors classical activation of macrophages and thus promotes inflammation.

## 4. Discussion

As expected, HFD increased most metabolic parameters in both WT and CKO mice. Notably, plasma free fatty acids and triglycerides were significantly increased in CKO mice on ND compared to those in WT mice on ND, supporting the notion that endogenous catalase may play an important role in basal lipid metabolism. Considering that adipose tissue and liver are the most important organs for lipid metabolism [36] and that catalase is highly expressed in WAT as well as liver, it is speculated that catalase deficiency in WAT and liver may play critical roles in dyslipidemia in CKO mice. CKO mice on HFD presented significantly increased markers of insulin resistance, such as fed and fasting plasma insulin and HOMA-IR. In line with our data, repeated administration of catalase conjugated to polyethylene glycol improves glucose tolerance and insulin sensitivity in obese mice [37]. On the other hand, triglycerides are esters of glycerol with three fatty acid groups and constitute the main chemical form by which vertebrates store and transport lipids in the body [38]. Increased level of triglycerides is the main feature of insulin resistance which results from increased oxidative stress in WAT and liver [32–34]. While the effect of catalase deficiency on the function of adipocytes and hepatocytes remains to be studied, our preliminary data suggest that catalase deficiency also accelerates high-fat diet-induced insulin resistance in WAT and liver (data not shown).

In adipose tissue, macrophages are major components of inflammation and insulin resistance [39]. Therefore, macrophage infiltration in epididymal WAT was estimated by mRNA and protein expression of the macrophage marker F4/80. Increased macrophage infiltration was observed in CKO mice compared to that in WT mice, regardless of diet. In addition, the number of crown-like structures, which are characteristic of M1 macrophages, was higher in CKO mice on HFD than in WT mice on the same diet, suggesting that endogenous catalase is key regulator of polarization in adipose tissue macrophages.

Therefore, we further investigated macrophage polarization in WAT, especially in the stromal vascular fraction, which contains preadipocytes, macrophages, T-cells, mast cells, and granulocytes, but not adipocytes. We found that protein and mRNA expression of CD11c, a major surface receptor marker of M1 macrophages [40], was significantly elevated in CKO mice on ND and HFD, along with levels of the proinflammatory cytokines iNOS and TNF-*α*, which are secreted from M1 macrophages [40]. In addition, PAI-1 mRNA levels were significantly increased in CKO mice. We note that PAI-1 has been suggested to facilitate the development of obesity and insulin resistance [41] and is upregulated especially in WAT macrophages of mice on HFD [42]. Indeed, PAI-1 has been used as an M1 marker in some studies, although it is not a specific M1 macrophage marker [43]. In contrast, CKO reduced protein and mRNA expression of CD206, a major receptor surface marker of M2 macrophages, regardless of diet. These results suggest that catalase deficiency induces a switch in macrophage polarization regardless of diet.

Confirming the reversal of CKO-mediated inflammation by catalase overexpression is a necessary strategy to provide proof-of-concept of direct role of catalase. The pharmacological and genetic approaches to study the role of catalase will complement each other. Due to our limitations in performing catalase overexpression into CKO macrophages, we have utilized 3-AT, a pharmacological inhibitor of catalase on the inflammatory response in RAW264.7 macrophages. Notably, the inhibitor induced inflammation, as assessed by increased JNK phosphorylation and mRNA expression of proinflammatory cytokines. We note that macrophages promote obesity-induced insulin resistance and inflammation through JNK expression [40]. In line with this result, siRNA knockdown of catalase also induced JNK phosphorylation (data not shown). Further, reduced catalase activity enhanced PA-induced inflammation and M1 polarization but suppressed M2 activation. Similarly, classically activated M1 macrophages were more abundant than M2 in primary bone marrow-derived macrophages genetically deficient in catalase, regardless of exposure to PA. Altogether, these data suggest that catalase regulates macrophage activation and preserves the balance between M1 and M2 macrophages.

Catalase is associated with various human diseases, including osteoarthritis, cancer, psoriasis and other skin disorders, ischemia-reperfusion injury, neurodegenerative disorders, and type 2 diabetes [44]. Catalase deficiency also stimulates fibronectin expression, accelerates diabetic kidney injury [27, 28], and increases mitochondrial reactive oxygen species, especially in Hs27 human diploid fibroblasts [45], presumably by increasing *β*-oxidation. Although we did not measure mitochondrial reactive oxygen species, these molecules are known to enhance classical M1 activation [46]. In addition, 3-AT elicits inflammatory responses in human alveolar macrophages by increasing basal H_2_O_2_ [30], highlighting the role of catalase in altering macrophage polarization to promote inflammation in adipose tissue.

In summary, we suggest that endogenous catalase plays an important role in the polarization of adipose tissue macrophages, both in basal conditions and under metabolic stress. Consequently, the enzyme inhibits inflammation and insulin resistance, as measured by fed and fasting insulin and by HOMA-IR. Thus, strategies based on catalase may be therapeutic against metabolic diseases.

## Supplementary Material

Supplementary figure 1. Catalase expresses in adipose tissue along with liver and kidney. Liver, kidney, heart, lung, brown adipose tissue (BAT), white adipose tissue (WAT), pancreas, spleen, testis and thyroid from wild type C57BL/6 J mice were subjected for catalase protein (obtained from Young In Frontier, Seoul, Korea) expression using western blotting analysis as described in method section.

## Figures and Tables

**Table 1:** Primer sequences of gene used in this study.

Gene name	Forward	Reverse
Arginase-1	5′-TGGCTTGCGAGACGTAGAC-3′	5′-CGTCAGGTGAATCGGCCTTT-3′
CD11c	5′-TGGCTTCAACTTGGATGCAG-3′	5′-CAACCACCACCCAGGAACTA-3′
CD206	5′-GTGGAGTGATGGAACCCCAG-3′	5′-CTGTCCGCCCAGTATCCATC-3′
F4/80	5′-CTGTAACCGGATGGCAAACT-3′	5′-ATGGCCAAGGCAAGACATAC-3′
IL-1*β*	5′-GTCAACGTGTGGGGGATGAA-3′	5′-AAGCAATGTGCTGGTGCTTC-3′
IL-6	5′-AGTTGCCTTCTTGGGACTGA-3′	5′-TCCACGATTTCCCAGAGAAC-3′
IL-10	5′-GCTCTTACTGACTGGCATGA-3′	5′-CGCAGCTCTAGCATGTG-3′
PAI-1	5′-AGGGCTTCATGCCCCACTTC-3′	5′-AGTAGAGGGCATTCACCAGC-3′
TNF-*α*	5′-CGTCAGCCGATTTGCTATCT-3′	5′-CGGACTCCGCAAAGTCTAAG-3′
18S	5′-CGAAAGCATTTGCCAAGAAT-3′	5′-AGTCGGCATCGTTTATGGTC-3′

**Table 2:** Metabolic characteristics of experimental mice.

Parameters	ND		HFD	
	WT	CKO	WT	CKO
Body weight (g)	32.2 ± 0.7	31.5 ± 0.9	45.1 ± 1.5^*∗*^	47.8 ± 2.2^#^
Epididymal fat mass (g)	0.38 ± 0.06	0.41 ± 0.10	1.94 ± 0.13^*∗*^	1.66 ± 0.13^#^
Plasma free fatty acids (*µ*M)	0.47 ± 0.02	0.6 ± 0.04^*∗*^	0.52 ± 0.07	0.77 ± 0.13^$^
Plasma triglycerides (mM)	0.72 ± 0.04	0.93 ± 0.07^*∗*^	0.97 ± 0.07^*∗*^	1.04 ± 0.11
Plasma LDL/VLDL (mg/dL)	21.5 ± 4.0	30.7 ± 1.5	70.9 ± 5.0^*∗*^	62.2 ± 8.6^#^
Plasma HDL (mg/dL)	89.7 ± 6.0	95.7 ± 4.9	90.7 ± 8.0	101.5 ± 5.5
Fed plasma insulin (ng/mL)	1.54 ± 0.38	2.22 ± 0.51	6.16 ± 1.11^*∗*^	10.08 ± 1.83^#,$^
Fasting plasma insulin (ng/mL)	0.21 ± 0.02	0.24 ± 0.06	0.50 ± 0.09^*∗*^	1.20 ± 0.29^#,$^
HOMA-IR	2.44 ± 0.34	2.22 ± 0.69	7.39 ± 1.24^*∗*^	14.17 ± 4.04^#,$^

WT and CKO C57BL/6 J mice were divided into four groups: WT ND, CKO ND, WT HFD, and CKO HFD. They were fed either ND (18% fat-derived calories) or HFD (60.3% fat-derived calories) for 21 weeks. Blood was collected from orbital sinus of all mice for measurement of plasma insulin at 21 weeks after HFD and a day before sacrificing. Data were shown as mean ± SE of 6–8 mice. ^*∗*^ *p* < 0.05 versus WT ND, ^#^ *p* < 0.05 versus CKO ND, and ^$^ *p* < 0.05 versus WT HFD.
