# Supplementary figures and images for: Novel Role of Endogenous Catalase in Macrophage Polarization in Adipose Tissue
Source: Mediators Inflamm. 2016 Aug 15;2016:8675905. doi: 10.1155/2016/8675905 (PMC5002490; doi:10.1155/2016/8675905)

**Catalase**

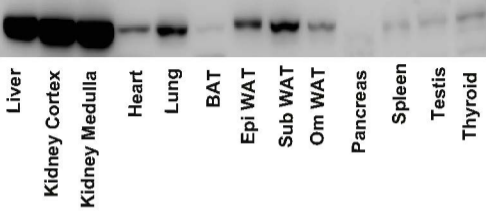

**Supplementary fig. 1**

Supplement: Supplementary file 1 — Supplementary figure 1. Catalase expresses in adipose tissue along with liver and kidney. Liver, kidney, heart, lung, brown adipose tissue (BAT), white adipose tissue (WAT), pancreas, spleen, testis and thyroid from wild type C57BL/6 J mice were subjected for catalase protein (obtained from Young In Frontier, Seoul, Korea) expression using western blotting analysis as described in method section. [file 8675905.f1.pdf]
